# Long non-coding RNA polymorphisms on 8q24 are associated with the prognosis of gastric cancer in a Chinese population

**DOI:** 10.7717/peerj.8600

**Published:** 2020-02-21

**Authors:** Yangyu Zhang, Yanhua Wu, Zhifang Jia, Donghui Cao, Na Yang, Yueqi Wang, Xueyuan Cao, Jing Jiang

**Affiliations:** 1 Division of Clinical Research, First Hospital of Jilin University, Changchun, Jilin, China; 2 Department of Gastric and Colorectal Surgery, First Hospital of Jilin University, Changchun, Jilin, China

**Keywords:** Gastric cancer, Genetic variation, lncRNA, 8q24, Prognosis

## Abstract

**Background:**

Gastric cancer (GC) remains the third leading cause of cancer death in China. Although genome-wide association studies have identified the association between several single nucleotide polymorphisms (SNPs) on 8q24 and the risk of GC, the role of these SNPs in the prognosis of GC in Chinese populations has not yet been fully evaluated. Therefore, this study was conducted to explore the association between long non-coding RNA (lncRNA) polymorphisms on 8q24 and the prognosis of GC.

**Methods:**

We genotyped 726 surgically resected GC patients to explore the association between eight SNPs in the lncRNAs CCAT1 (rs10087719, rs7816475), PCAT1 (rs1026411), PRNCR1 (rs12682421, rs13252298), and CASC8 (rs1562430, rs4871789, rs6983267) transcribed from the 8q24 locus and the prognosis of GC in a Chinese population.

**Results:**

We found that the patients carrying rs12682421 AA genotypes survived for a shorter time than those with the GG/GA genotype (HR = 1.39, 95% confidence interval (CI) [1.09–1.78]). Compared with the CC/CT genotype, the TT genotype of rs1562430 was associated with an increased risk of death (HR = 1.38, 95% CI [1.06–1.80]). Furthermore, the results also identified the rs1026411 SNP as an independent prognostic factor for poor survival in GC patients. Patients carrying AA/AG variant genotypes had a 36% increased risk of death compared to those carrying the GG genotype (HR = 1.36, 95% CI [1.06–1.74]). These findings suggested that the rs12682421, rs1026411 and rs1562430 SNPs may contribute to the survival of GC and be prognostic markers for GC.

## Introduction

Gastric cancer (GC) is the fifth most common malignancy and the third leading cause of cancer-related mortality in the world. A total of 1,033,701 new cases of GC were estimated to have occurred in 2018 (Bray et al., 2018). Although the incidence of GC has been declining in the last decades in most regions, it remains a common cancer among many populations in East Asia. Due to a high incidence rate and a large population, more than 40% of GC cases worldwide have occurred in China, according to GLOBOCAN 2012 (Ferlay et al., 2015). In the last decade, the mortality rate of GC has declined conspicuously due to the improved treatment approaches, but the prognosis of GC is still poor; the 5-year survival rate is 29.0% (Zeichner et al., 2017).

Regarding treatment approaches, tumorectomy with adjuvant or neoadjuvant chemotherapy and radiotherapy are the most effective treatments for GC. However, despite improvements in surgical and adjuvant multimodal treatments, the prognosis of GC is still poor due to late diagnosis and extreme intra- and inter-tumour heterogeneity (Bonelli et al., 2019). The heterogeneity makes the selection of treatment options difficult, and previous studies have found that patients with the same pathological stage and tumour grade who receive similar therapies may have different clinical outcomes, a finding that indicates the significance of individual variants influenced by genetic and environmental factors (Wang et al., 2018). Therefore, exploring genetic variations in key genes involved in tumour progression as biomarkers to improve the prognosis prediction of GC patients is imperative.

The results from genome-wide association studies (GWASs) have also identified single nucleotide polymorphisms (SNPs), the most common type of genetic variations in the human genome, in relation to the tumourigenesis of GC (Abnet et al., 2010; Sakamoto et al., 2008; Shi et al., 2011). GWASs have identified several loci, including 1q22, 5p13.1 and 8q24, that are associated with GC susceptibility, mainly in populations in Asia (Saeki et al., 2013; Shi et al., 2011; Wadhwa et al., 2013). Particularly, a series of evidence has suggested that 8q24 chromosome region can not only affect GC susceptibility (Zhi, Shi & Liu, 2017), but also confer GC patients with different prognosis (Ma et al., 2015; Wang et al., 2016), which further verified that genetic background play an important role in gastric carcinogenesis and progression.

Although paying attention to known genes might generate further understanding in development and therapy of GC, newly-developed markers such as long non-coding RNAs (lncRNAs) may lead novel insight into the mechanism of GC risk or treatment. LncRNAs are noncoding transcripts that are more than 200 nucleotides long. Although they were initially regarded as ‘transcriptional noise’, increasing studies have found that lncRNAs can regulate local or global gene expression through transcriptional, post-transcriptional and epigenetic regulation (Mercer, Dinger & Mattick, 2009). As lncRNAs play multiple roles in the regulation of gene expression, aberrant lncRNA expression may therefore occur during carcinogenesis and disease development. These advantages make lncRNAs potential biomarkers for the diagnosis, prognosis and therapy of a variety of cancers, including GC (Wu & Hsieh, 2019; Yuan et al., 2018; Zhao et al., 2015).

The 8q24 chromosome region has been reported to express several lncRNAs in different human tumours (Huang, Zhang & Shao, 2018; Tong et al., 2018; Xiang et al., 2014). The association between polymorphisms in lncRNAs and the risk of GC has been studied in several ethnicities (Labrador et al., 2015; Pan et al., 2016; Ülger et al., 2017). However, there are few studies that have investigated the prognostic value of lncRNA polymorphisms on 8q24 in GC patients. Hence, this study was performed to examine whether variants of lncRNA colon cancer-associated transcript (CCAT1), prostate cancer-associated transcript 1 (PCAT1), prostate cancer non-coding RNA 1 (PRNCR1) and cancer susceptibility candidate 8 (CASC8) genes on chromosome 8q24 are associated with survival in a Chinese population with GC. It may bring benefits for individualised treatment and consequently improve survival outcomes.

## Materials and Methods

### Study population

Subjects of the study were the newly diagnosed GC patients recruited from the Department of Gastric and Colorectal Surgery of the First Hospital of Jilin University from 2008 to 2013. A total of 756 patients who underwent tumourectomy without receiving chemotherapy or radiotherapy before surgery were enrolled in this study. The individual characteristics (gender, age) and clinical data (tumour size, histological type, histological grade, lymph metastasis, distant metastasis, depth of invasion, neural invasion and therapy) were collected from the medical records. TNM classification, based on the 2010 seventh edition of the American Joint Committee on Cancer (AJCC) guidelines(Washington, 2010), was used to evaluate the clinical stage of the cancer. The evaluation of *Helicobacter pylori* infection was performed via a serum immunoglobulin G (IgG) antibody test by an enzyme-linked immunosorbent assay (ELISA) using an *H. pylori*-IgG ELISA kit (Biohit, Helsinki, Finland). Postoperative chemotherapy was identified as an effective therapy for at least three cycles.

### Ethics statement

Each patient in this study signed an informed consent form before sample and information collection. This study was approved by the ethics committees of the First Hospital of Jilin University (2013-005).

### Follow-up

The follow-up of the patients was conducted 3 months, 6 months, and 1 year after surgery and every 1 year thereafter until the death of the patient or loss to follow-up. The data from each follow-up visit were collected. Subjects were excluded if they were lost to follow-up at the first phone interview or died due to complications of the surgery during the perioperative period (within 30 days after surgery). The survival time was considered as the duration (i) from the date of the surgery to the date of death if the GC patient had died or (ii) from the date of the surgery to the date of the last phone interview if the patient was lost to follow-up or to the end of the study if the patient was still alive.

### Tagging SNP selection

From whole blood sample of each patient, we extracted genomic DNA using a MagPure Tissue and Blood DNA KF Kit (Magen, Guangzhou, China). The tag SNPs and the well-studied SNPs on 8q24 that were previously reported be associated with gastrointestinal tumours were selected. These SNPs included CCAT1 rs10087719, CCAT1 rs7816475, PCAT1 rs1026411, PRNCR1 rs12682421, PRNCR1 rs13252298, CASC8 rs1562430, CASC8 rs4871789 and CASC8 rs6983267. The SNPinfo (http://snpinfo.niehs.nih.gov/), GVS (http://gvs.gs.washington.edu/GVS147/) and F-SNP (Lee & Shatkay, 2008) databases were used to select tag SNPs. The minor allele frequency of all the SNPs was > 0.05 based on the Han Chinese Population.

### Genotyping

Single nucleotide polymorphism genotyping was conducted by the MassARRAY technology platform (Sequenom, CA, USA) and was determined by the Bio Miao Biological Technology Co., Ltd. (Beijing, China). The detection rates for rs10087719, rs7816475, rs1026411, rs12682421, rs13252298, rs1562430, rs4871789 and rs6983267 were 100%, 98%, 99%, 100%, 100%, 100%, 98%, and 94%, respectively. The linkage disequilibrium (LD) was established with a threshold of the pairwise *r*^2^ coefficient greater than 0.80 and the extent of LD between the eight SNPs were estimated using Haploview 4.2 (Broad Institute of MIT and Harvard, Cambridge, MA, USA). None of the eight SNPs were located at CCAT1, PRNCR1 or CASC8 with LD.

### Transcription factor binding site prediction

Transcription factor binding sites (TFBS) were predicted using the PROMO database (http://alggen.lsi.upc.es/cgi-bin/promo_v3/promo/promoinit.cgi?dirDB=TF_8.3).

### Statistical analysis

Frequency and proportion were used to describe the categorical variables. A goodness of fit χ^2^ test was used to test the Hardy-Weinberg equilibrium (HWE) of each SNP. Survival curves of the GC patients based on each SNP were plotted by the Kaplan–Meier method and were compared by log-rank test. The Cox regression model was used to calculate hazard ratios (HRs) with 95% confidence intervals (CIs) and to evaluate the associations between genotypes of each SNP and overall survival after adjusting for potential confounders (age, gender, *H. pylori*, tumour size, TNM stage, histological type, histological grade, chemotherapy, lymph vascular invasion and neural invasion). We used the Bonferroni correction method to adjust for multiple testing with a significance threshold set at *P* = 0.00625 (0.05/8 SNPs). All statistical analyses were performed using the SPSS 21.0 software (IBM SPSS, IBM Corp, Armonk, NY, USA).

## Results

### Characteristics of patients

**Figure 1:**
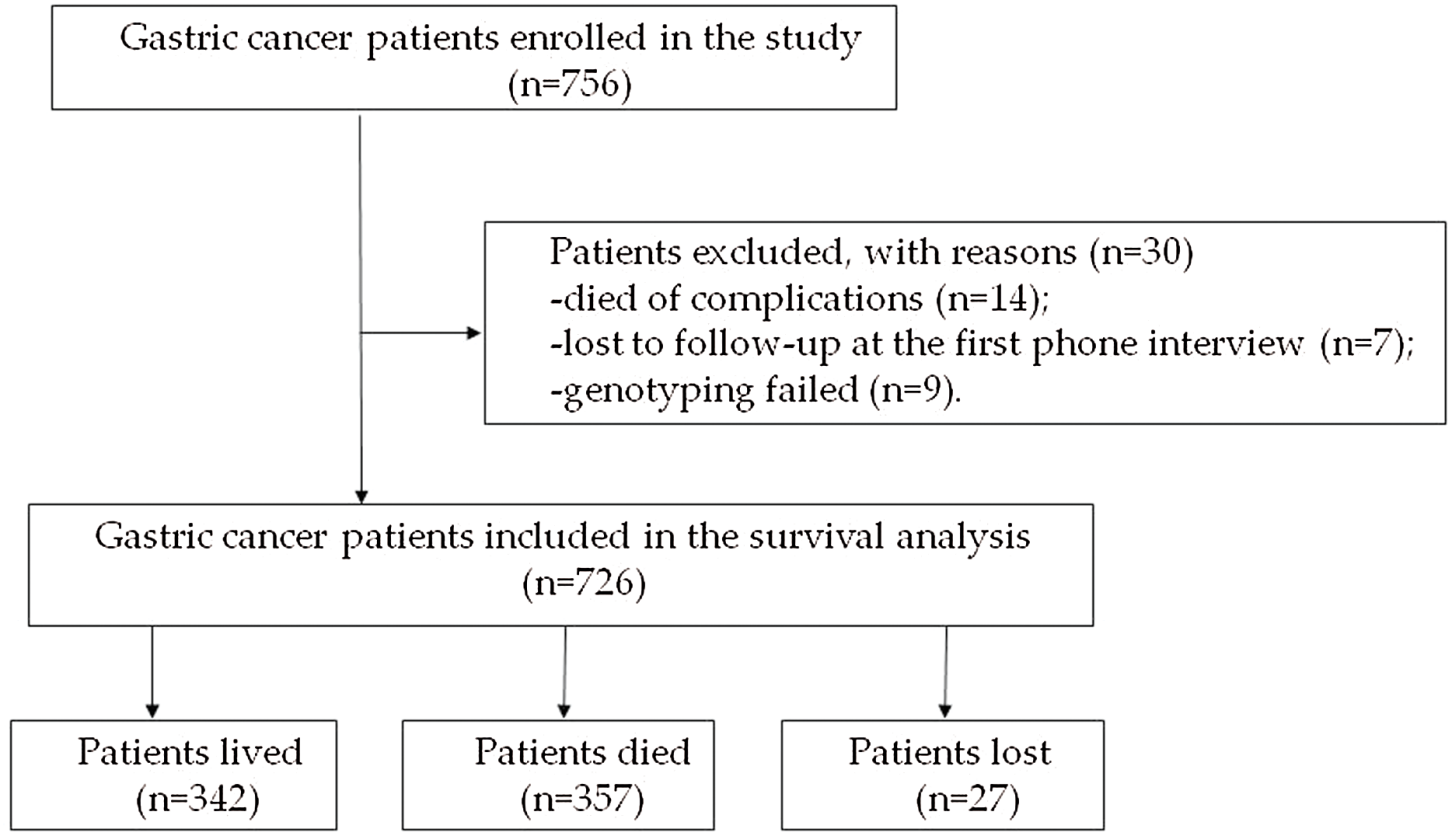
Flow chart of the enrolled subjects.

A total of 756 diagnosed GC patients were enrolled in the study. Fourteen patients died of complications from the surgery during the preoperative period, seven patients were lost to follow-up at the first phone interview and the genotyping of nine patients failed. The remaining 726 patients were included in the study for the subsequent analysis. At the end of the study, 27 patients were lost to follow-up, 357 patients died, and 342 patients were alive (Fig. 1). The duration of follow-up was from 1 month to 109 months, and the median follow-up time was 70.7 months. The characteristics of the 726 patients are shown in Table 1.

**Table 1:** Characteristics of the GC patients.

Variables		*N*	%
Age (years)	<60	340	46.8
	≥60	386	53.2
Gender	Male	547	75.3
	Female	179	24.7
Smoking	Yes	281	39.0
	No	440	61.0
Drinking	Yes	198	27.4
	No	524	72.6
Family history	Yes	47	6.5
	No	672	93.5
*H. pylori*	Positive	438	68.7
	Negative	200	31.3
Tumour size	<5 cm	420	57.9
	≥5 cm	306	42.1
TNM stage	I	129	17.8
	II	271	37.3
	III	326	44.9
Histological type	Tubular	571	78.6
	Signet-ring cell	68	9.4
	Other	87	12.0
Histological grade	Low-grade	218	30.0
	High-grade	508	70.0
Lymph vascular invasion	Yes	514	70.8
	No	212	29.2
Neural invasion	Yes	399	55.0
	No	327	45.0
Chemotherapy	Yes	314	43.3
	No	412	56.7

### Genotype and allele frequencies of the eights SNPs

The genotype frequencies of seven SNPs (rs10087719, rs7816475, rs1026411, rs12682421, rs13252298, rs1562430, rs4871789) in the subjects were in HWE with non-significant χ^2^ values (*P* > 0.05). The rs6983267 locus, however, was found to deviate from the HWE (*P* < 0.001). We randomly selected 10% of the samples for repeat genotyping of rs6983267, but the result remained the same. The distributions of the genotype and allele frequencies of the eight SNPs in subjects are shown in Table 2. After Bonferroni correction for multiple testing, none of the eight SNPs were significantly associated with the survival of GC. However, there was a statistically significant tendency for rs12682421 (log-rank *P* = 0.03). As shown in Table 2, patients with the rs12682421 GA genotype tended to have a better prognosis (HR = 0.77, 95% CI [0.61–0.97]) than patients with the AA genotype.

**Table 2:** Distributions of the genotypes of the gastric cancer patients.

Gene	Genotypes		Patients, *N*	Death, *N* (%)	MST	HR (95% CI)	Log rank *P*
CCAT1	rs10087719	AA	495	250 (50.50)	64.89	1.00	0.73
		AG	209	99 (47.37)	75.70	0.93 [0.74–1.18]	
		GG	20	8 (40.00)	54.67*	0.81 [0.40–1.63]	
CCAT1	rs7816475	GG	572	280 (48.95)	70.04	1.00	0.65
		AG	134	69 (51.49)	56.38	1.08 [0.83–1.41]	
		AA	6	2 (33.33)	55.99*	0.61 [0.15–2.45]	
PCAT1	rs1026411	GG	257	120 (46.69)	75.20	1.00	0.32
		AG	353	181 (51.27)	60.85	1.20 [0.95–1.50]	
		AA	111	53 (47.75)	63.07*	1.11 [0.80–1.53]	
PRNCR1	rs12682421	AA	443	230 (51.92)	58.51	1.00	0.03
		GA	248	106 (42.74)	68.84*	0.77 [0.61–0.97]	
		GG	33	21 (63.64)	37.78	1.25 [0.80–1.95]	
PRNCR1	rs13252298	AA	330	158 (47.88)	70.05	1.00	0.75
		AG	315	157 (49.84)	69.29	1.09 [0.87–1.36]	
		GG	79	42 (53.16)	55.39	1.07 [0.76–1.51]	
CASC8	rs1562430	TT	505	257 (50.89)	64.30	1.00	0.17
		CT	199	93 (46.73)	79.05	1.13 [0.84–1.53]	
		CC	19	6 (31.58)	75.20	0.68 [0.35–1.16]	
CASC8	rs4871789	GG	251	129 (51.39)	64.89	1.00	0.64
		AG	344	165 (47.97)	73.50	0.90 [0.71–1.13]	
		AA	119	58 (48.74)	67.35	0.91 [0.67–1.24]	
CASC8	rs6983267	TT	172	89 (51.74)	65.68	1.00	0.59
		GT	416	195 (46.88)	79.05	1.01 [0.82–1.24]	
		GG	95	48 (50.53)	67.35	0.93 [0.84–1.09]	

**Notes:**

MST, median survival time, months.

* Mean survival time was provided when MST could not be calculated.

### Multivariate Cox regression analysis of SNPs and gastric cancer survival

**Figure 2:**
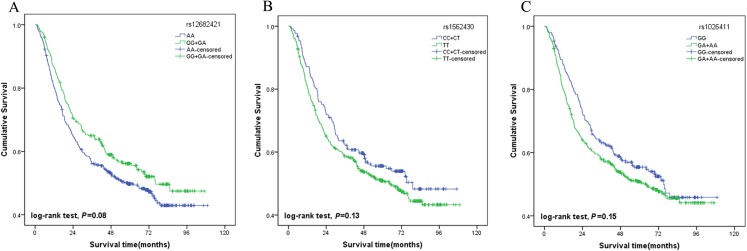
Association of genotypes with overall survival in gastric cancer patients. (A) Plot for rs12682421 using the dominant model (GG/GA vs. AA); (B) Plot for rs1562430 using the dominant model (CC/CT vs. TT); (C) Plot for rs1026411 using the dominant model (AA/AG vs. GG).

A multivariate stepwise Cox regression model was performed to explore the independent prognostic factor for GC. The results showed that tumour size, TNM stage, lymph vascular invasion and chemotherapy were associated with the survival of the patients. The AA genotypes of rs12682421 were associated with a significantly increased risk of death compared with that of the GG/GA genotype (HR = 1.39, 95% CI [1.09–1.78]). The TT genotypes of rs1562430 were also associated with increased risk of death compared with that of the CC/CT genotype (HR = 1.38, 95% CI [1.06–1.80]). The results also identified rs1026411 SNP as an independent prognostic factor for the poor survival of GC patients; patients carrying AA/AG genotypes had a 36% increased risk of death compared to those carrying the GG genotype (HR = 1.36, 95% CI [1.06–1.74]) (Table 3). The survival curves of the three SNPs are shown in Fig. 2.

**Table 3:** Stepwise Cox regression analysis of gastric cancer survival. Age, gender, *H. pylori*, tumour size, TNM stage, histological type, histological grade, chemotherapy, lymph vascular invasion, neural invasion and eight SNPs polymorphism were used as variables in the regression model.

Genotypes	*P*	Adjusted HR (95% CI)
Tumour size	0.005	1.42 [1.11–1.81]
Lymph vascular invasion	<0.001	2.09 [1.45–3.02]
TNM stage		
II vs. I	0.007	2.25 [1.24–4.07]
III vs. I	<0.001	6.83 [3.77–12.36]
Chemotherapy	0.007	0.72 [0.56–0.91]
rs12682421 (AA vs. GA+GG)	0.009	1.39 [1.09–1.78]
rs1562430 (TT vs. CC+CT)	0.016	1.38 [1.06–1.80]
rs1026411 (AA+AG vs. GG)	0.017	1.36 [1.06–1.74]

### Stratified analysis of the genotypes associated with gastric cancer prognosis

**Figure 3:**
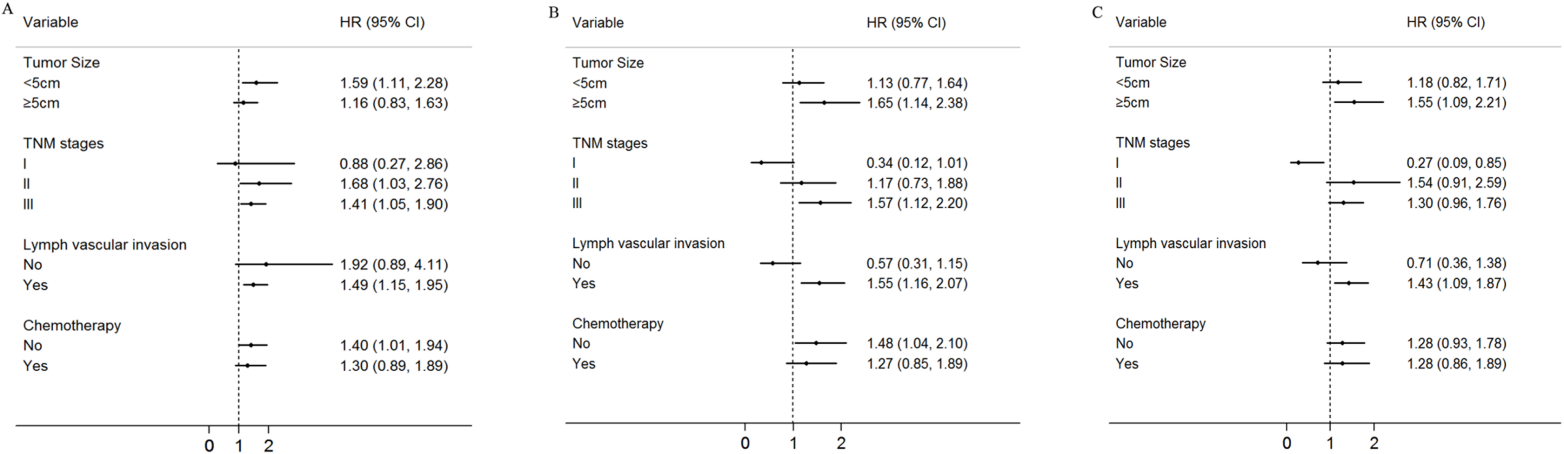
Stratified analysis for rs12682421, rs1562430 and rs1026411genotypes associated with gastric cancer patients’ survival. Age, gender, *H*. *pylori*, tumour size, TNM stage, histological type, histological grade, chemotherapy, lymph vascular invasion, neural invasion and eight SNP polymorphisms were used as variables in the regression model. (A) Stratified analysis of rs12682421 genotypes associated with gastric cancer patients’ survival (AA vs. GA+GG); (B) Stratified analysis of rs1562430 genotypes associated with gastric cancer patients’ survival (TT vs. CC+CT); (C) Stratified analysis of rs1026411 genotypes associated with gastric cancer patients’ survival (AA+AG vs. GG).

Moreover, the associations between the three SNPs (rs12682421, rs1562430, rs1026411) and the survival of the GC patients was evaluated by a stratified analysis of tumour size, TNM stage, lymph vascular invasion and chemotherapy. Compared to patients with the GA/GG genotype of rs12682421, patients with the AA variant genotype had a higher death risk in the subgroup of patients with tumour sizes <5 cm (HR = 1.59, 95% CI [1.11–2.28]), an advanced TNM stage (TNM stage II: HR = 1.68, 95% CI [1.03–2.76]; TNM stage III: HR = 1.41, 95%CI [1.05–1.90]), lymph vascular invasion (HR = 1.49, 95% CI [1.15–1.95]) or no postoperative chemotherapy(HR = 1.40, 95% CI [1.01–1.94]) (Fig. 3A). Compared to patients with the CC/CT genotype, patients carrying TT genotypes of rs1562430 had a higher death risk in the subgroup of patients with tumour sizes ≥5 cm (HR = 1.65, 95% CI [1.14–2.38]), TNM stage III (HR = 1.57, 95% CI [1.12–2.20]), or lymph vascular invasion (HR = 1.55, 95%CI [1.16–2.07]) and in the subgroup of patients who did not receive postoperative chemotherapy (HR = 1.48, 95% CI [1.04–2.10]) (Fig. 3B). Compared to patient with the GG genotype of rs1026411, patients with the AA/AG variant genotype had a higher death risk in the subgroup of patients with tumour sizes ≥5 cm (HR = 1.55, 95% CI [1.09–2.21]) or lymph vascular invasion (HR = 1.43, 95% CI [1.09–1.87]) (Fig. 3C).

## Discussion

Clinically, therapeutic decision making and prognostic prediction for GC patients still depend on the TNM staging system. However, due to the significant heterogeneity within the same stage, the TNM stage alone is not sufficient to predict the prognosis of GC. More significantly, although the TNM staging system classifies patients into subgroups with different clinical outcomes, it provides limited information about therapeutic effects in individual patients (Li et al., 2017; Qu et al., 2015). Therefore, it is crucial and necessary to identify new biomarkers for GC patients to complement the TNM staging system in order to improve the prediction of prognosis and guide therapeutic decisions. Our study focused on the investigation of eight lncRNA SNPs on 8q24 that predispose GC patients to survival. Multivariate analysis revealed that the AA genotype of rs12682421, the TT genotype of rs1562430 and the AA/AG genotype of rs1026411 could serve as potential markers to predict the unfavourable survival of GC patients in the Chinese population. Furthermore, the prominent prognostic effect of the three SNPs was more evident in advanced subgroups of GC patients.

Recent research has suggested that lncRNAs, as oncogenes or tumour suppressor genes, could be involved in the development of cancer and be associated with tumour metastasis and prognosis (Song et al., 2017; Weidle et al., 2017). Given that the majority of the GWAS-identified cancer risk SNPs are located in the noncoding region, the expression and function of lncRNAs are more likely to be impacted by the SNPs (Gao & Wei, 2017). Moreover, GWAS have identified 8q24 as a hotspot for cancer-associated SNPs on account of the strength, density and high allele frequency of these SNPs (Sur et al., 2013). Although several studies have revealed that lncRNAs on 8q24, including PRNCR1, CCAT2 and PCAT1, encompass the cancer predisposition SNPs (Guo et al., 2019; Ling et al., 2013; Wang & Wang, 2019; Yang et al., 2019), the prognostic significance of these lncRNAs in GC patients has not yet been fully explored.

Prostate cancer non-coding RNA 1, a lncRNA transcribed from 8q24, participates in the carcinogenesis of prostate cancer (PCa) by activating androgen receptor (AR) (Chung et al., 2011); in addition, polymorphisms of the lncRNA PRNCR1 were noted in many cancers, including colorectal cancer (AlMutairi & Parine, 2019), prostate cancer (Sattarifard et al., 2017), and GC (He et al., 2017). A meta-analysis conducted by Huang, Zhang & Shao (2018) showed that rs16901946 of PRNCR1, which was in complete LD with rs12682421, was associated with an increased risk of GC in the dominant model. A study performed by He et al. (2017) that aimed to assess the GC susceptibility and GC prognostic value of the polymorphisms in PRNCR1, found that rs16901946 G allele carriers (linked with rs12682421 G allele) have an increased risk of GC, but this polymorphism did not exhibit any significant prognostic value for GC. However, in our study, we found that compared with the GA/GG genotype, the PRNCR1 rs12682421 AA genotype was a poor prognostic factor for GC, which is different from the results of the study by He et al. We considered that the reasons for the inconsistent conclusion may be due to the fact that doctor He’s study has a smaller sample size (*N* = 494), a shorter follow-up time (the patients were followed for up to 4 years) and fewer events compared with our study.

Rs1562430 is located in the intron of CASC8, a long noncoding RNA (lncRNA), and overlaps with the POU5F1B gene. Previous studies have revealed that the rs1562430 SNP has a strong association with the risk of breast cancer and colorectal cancer (He et al., 2011; Kim et al., 2012; Silvestri et al., 2015). Ma et al. (2015) found that there were no associations between the rs1562430 genotype and the survival of GC patients in a Chinese population. However, in the present study, we found that rs1562430 TT was associated with a significantly lower survival rate in GC patients than CC/CT. The study conducted by Ma et al. included patients with TNM stage IV, and the histological type of 42.5% of patients was intestinal. Conversely, our study excluded patients with TNM stage IV, and more than 70% of patients exhibited an intestinal histological type. Moreover, nearly 70% of the patients in our study were infected with *H. pylori*. Therefore, the differences in the pathogenic environment and genetic background of the patients included in the two studies may be the reasons for the inconsistent results.

Existing studies have found that PCAT1 overexpression occurred in PCa, lung cancer and colorectal cancer (Ge et al., 2013; Prensner et al., 2011; Zhao, Hou & Zhan, 2015). Shi et al. (2015) identified that ESCC patients with high levels of PCAT1 had poorer survival times than those with low levels of PCAT1. Moreover, recent studies have suggested that a PCAT1 genetic variant may play an essential role in the susceptibility to several cancers (Ren et al., 2017; Zhao, Hou & Zhan, 2015). Yuan et al. (2018) found that rs1902432 in PCAT1 was significantly associated with an increased risk of PCa, and Lin et al. (2017) found that rs710886 of PCAT1 was significantly associated with bladder cancer risk in a Chinese population. As far as we know, no study has been conducted on the role of PCAT1 polymorphisms in the prognosis of GC. In the present study, we found that, compared to GG, rs1026411 AA/AG was associated with a poor prognosis of GC patients (HR = 1.33, 95% CI [1.03–1.70]).

Previous studies have found that SNPs in lncRNAs have different prognostic values when they occur with different clinical features (Ma et al., 2015; Xiong et al., 2017); therefore, we conducted a stratified analysis by tumour size, TNM stage, lymph vascular invasion and chemotherapy. The unfavourable prognostic effects of rs1026411, rs1562430 and rs12682421 were more evident among patients with increased TNM stage and lymph vascular invasion, which indicated that these three SNPs may have higher predictive value in advanced stages of GC. Previous studies have demonstrated that genomic polymorphisms can affect drug transport, metabolism and cellular response and cause individual variations in terms of the response and even overall survival (Ulrich, Robien & McLeod, 2003). Increasing evidence has also suggested that SNPs in some lncRNAs are related to chemotherapy response and could provide effective therapeutic targets for GC treatment (Ozgur et al., 2019; Zhang & Du, 2016). Our results showed that in patients who did not receive chemotherapy, the rs12682421 AA genotype and rs1562430 TT genotype could predict poor survival (HR = 1.40, 95% CI [1.01–1.94]; HR = 1.48, 95% CI [1.04–2.10]); however, in the subgroup of patients who received chemotherapy, the difference in the genomic polymorphisms of the two SNPs disappeared. The effect of the genetic background of the two SNPs may be overshadowed by the advantages of chemotherapy, considering that the multivariate analysis results showed that patients with postoperative chemotherapy tended to have a favourable prognosis (HR = 0.72, 95% CI [0.56–0.91]).

Because lncRNAs could play a crucial role in the regulation of gene expression via transcription and transcription factors often play important roles in tumourigenesis, we used bioinformatics data from the PROMO TFBS database to predict the possible functions of rs12682421, rs1562430 and rs1026411. We found that the C allele at the rs1562430 locus allowed binding to the glucocorticoid receptor α (GRα), which is a transcription factor that increases genes that participate in cell cycle arrest and apoptosis (Kumar, Johnson & Thompson, 2004; Patki & McFall, 2018; Yemelyanov et al., 2006). GRα could be bound and activated by glucocorticoids, and previous studies have shown that a higher expression of glucocorticoids receptors has been correlated with a better prognosis in bladder cancer (Ishiguro et al., 2014; Zheng et al., 2012). Therefore, we considered that the rs1562430 T allele may be associated with a lower GRα expression, which affects the prognosis of the GC patients. Additionally, the A allele at the rs12682421 locus was found to be allowed binding to the GRβ transcription factor, which is a different isoform of GR. GRβ lacks the ligand-binding domain for glucocorticoids (Hinds et al., 2010) and has been indicated to inhibit GRα (Leung et al., 1997; Hinds et al., 2010; Kubin et al., 2016). GRβ has been demonstrated to be involved in the migration of bladder cancer and brain cancer (Mcbeth et al., 2016; Ying et al., 2013), and some other studies have also reported that GRβ levels are elevated in inflammatory diseases and cancers, leading to increased progression (Zhu et al., 2007; Marino et al., 2016; Psarra et al., 2005). Hence, we hypothesise that the poor prognosis of patients with the rs12682421 A allele may be associated with a higher GRβ expression. Moreover, we found that the G allele at the rs1026411 locus facilitated binding to the polyomavirus enhancer activator 3 (PEA3) transcription factor, which belongs to the PEA3 subfamily within the E-twenty-six domain transcription factor superfamily (Kandemir et al., 2017). Members of the PEA3 subfamily have been demonstrated in previous studies be associated with a variety of cancers (Cowden Dahl, Zeineldin & Hudson, 2007; Keld et al., 2010; Kim et al., 2015), but a study conducted by Keld et al. (2011) showed that PEA3 upregulation in isolation does not predict prognosis in any stage of GC. The specific roles of GRα, GRβ and PEA3 in GC should be verified in further studies.

There are several limitations in present study that should be noted. First, although the median follow-up time was 70.7 months, more than half of the patients survived, and the number of events was insufficient, which may limit the statistical power of our findings. Second, despite the fact that we found associations between three SNPs and overall survival of GC, the mechanisms are still not clear and need to be further elucidated. Third, our study was based on a single group of patients. Hence, other independent replications and multi-centre studies need to be done to explore the role of genetic polymorphisms of lncRNA on 8q24 in the prognosis of GC in different populations.

## Conclusions

In summary, the present study revealed that the PRNCR1 rs12682421 AA genotype, the CASC8 rs1562430 TT genotype and the PCAT1 rs1026411 AA/AG genotype could serve as potential markers to predict the unfavourable survival of GC patients in the Chinese population. These three SNPs may be used as prognostic markers in combination with traditional clinical prognostic factors to refine therapeutic decisions for the individualised treatment of GC.

## Supplemental Information

10.7717/peerj.8600/supp-1 Supplemental Information 1 Raw data.

10.7717/peerj.8600/supp-2 Supplemental Information 2 Codebook of the categorical data.

## Additional Information and Declarations

### Data Availability

The following information was supplied regarding data availability: The raw measurements are available in the Supplemental Files.

## References

[ref-1] Abnet CC, Freedman ND, Hu N, Wang Z, Yu K, Shu X-O, Yuan J-M, Zheng W, Dawsey SM, Dong LM, Lee MP, Ding T, Qiao Y-L, Gao Y-T, Koh W-P, Xiang Y-B, Tang Z-Z, Fan J-H, Wang C, Wheeler W, Gail MH, Yeager M, Yuenger J, Hutchinson A, Jacobs KB, Giffen CA, Burdett L, Fraumeni JF, Tucker MA, Chow W-H, Goldstein AM, Chanock SJ, Taylor PR (2010). A shared susceptibility locus in PLCE1 at 10q23 for gastric adenocarcinoma and esophageal squamous cell carcinoma. Nature Genetics.

[ref-2] AlMutairi M, Parine NR (2019). Association between polymorphisms in PRNCR1 and risk of colorectal cancer in the Saudi population. PLOS ONE.

[ref-3] Bonelli P, Borrelli A, Tuccillo FM, Silvestro L, Palaia R, Buonaguro FM (2019). Precision medicine in gastric cancer. World Journal of Gastrointestinal Oncology.

[ref-4] Bray F, Ferlay J, Soerjomataram I, Siegel RL, Torre LA, Jemal A (2018). Global cancer statistics 2018: GLOBOCAN estimates of incidence and mortality worldwide for 36 cancers in 185 countries. CA: A Cancer Journal for Clinicians.

[ref-5] Chung S, Nakagawa H, Uemura M, Piao L, Ashikawa K, Hosono N, Takata R, Akamatsu S, Kawaguchi T, Morizono T, Tsunoda T, Daigo Y, Matsuda K, Kamatani N, Nakamura Y, Kubo M (2011). Association of a novel long non-coding RNA in 8q24 with prostate cancer susceptibility. Cancer Science.

[ref-6] Cowden Dahl KD, Zeineldin R, Hudson LG (2007). PEA3 is necessary for optimal epidermal growth factor receptor-stimulated matrix metalloproteinase expression and invasion of ovarian tumor cells. Molecular Cancer Research.

[ref-7] Ferlay J, Soerjomataram I, Dikshit R, Eser S, Mathers C, Rebelo M, Parkin DM, Forman D, Bray F (2015). Cancer incidence and mortality worldwide: sources, methods and major patterns in GLOBOCAN 2012. International Journal of Cancer.

[ref-8] Gao P, Wei GH (2017). Genomic insight into the role of lncRNAs in cancer susceptibility. International Journal of Molecular Sciences.

[ref-9] Ge X, Chen Y, Liao X, Liu D, Li F, Ruan H, Jia W (2013). Overexpression of long noncoding RNA PCAT-1 is a novel biomarker of poor prognosis in patients with colorectal cancer. Medical Oncology.

[ref-10] Guo Q, Lv S, Wang B, Li Y, Cha N, Zhao R, Bao W, Jia B (2019). Long non-coding RNA PRNCR1 has an oncogenic role in breast cancer. Experimental and Therapeutic Medicine.

[ref-11] He BS, Sun HL, Xu T, Pan YQ, Lin K, Gao TY, Zhang ZY, Wang SK (2017). Association of genetic polymorphisms in the lncRNAs with gastric cancer risk in a Chinese population. Journal of Cancer.

[ref-12] He J, Wilkens LR, Stram DO, Kolonel LN, Henderson BE, Wu AH, Le ML, Haiman CA (2011). Generalizability and epidemiologic characterization of eleven colorectal cancer GWAS hits in multiple populations. Cancer Epidemiology, Biomarkers & Prevention.

[ref-13] Hinds TD, Sadeesh R, Cash HA, Stechschulte LA, Garrett H, Najjar SM, Sanchez ER (2010). Discovery of glucocorticoid receptor-β in mice with a role in metabolism. Molecular Endocrinology.

[ref-14] Huang X, Zhang W, Shao Z (2018). Association between long non-coding RNA polymorphisms and cancer risk: a meta-analysis. Bioscience Reports.

[ref-15] Ishiguro H, Kawahara T, Netto G, Miyamoto H (2014). MP50-15 reduced glucocorticoid receptor expression predicts bladder tumor recurrence and progression. Journal of Urology.

[ref-16] Kandemir B, Dag U, Bakir Gungor B, Durasi IM, Erdogan B, Sahin E, Sezerman U, Aksan Kurnaz I (2017). In silico analyses and global transcriptional profiling reveal novel putative targets for Pea3 transcription factor related to its function in neurons. PLOS ONE.

[ref-17] Keld R, Guo B, Downey P, Cummins R, Gulmann C, Ang YS, Sharrocks AD (2011). PEA3/ETV4-related transcription factors coupled with active ERK signalling are associated with poor prognosis in gastric adenocarcinoma. British Journal of Cancer.

[ref-18] Keld R, Guo B, Downey P, Gulmann C, Ang YS, Sharrocks AD (2010). The ERK MAP kinase-PEA3/ETV4-MMP-1 axis is operative in oesophageal adenocarcinoma. Molecular Cancer.

[ref-19] Kim HJ, Kim SH, Yu EJ, Seo WY, Kim JH (2015). A positive role of DBC1 in PEA3-mediated progression of estrogen receptor-negative breast cancer. Oncogene.

[ref-20] Kim H-C, Lee J-Y, Sung H, Choi J-Y, Park SK, Lee K-M, Kim YJ, Go MJ, Li L, Cho YS, Park M, Kim D-J, Oh JH, Kim J-W, Jeon J-P, Jeon S-Y, Min H, Kim HM, Park J, Yoo K-Y, Noh D-Y, Ahn S-H, Lee MH, Kim S-W, Lee JW, Park B-W, Park W-Y, Kim E-H, Kim MK, Han W, Lee S-A, Matsuo K, Shen C-Y, Wu P-E, Hsiung C-N, Lee J-Y, Kim H-L, Han B-G, Kang D (2012). A genome-wide association study identifies a breast cancer risk variant in ERBB4 at 2q34: results from the Seoul Breast Cancer Study. Breast Cancer Research.

[ref-21] Kubin ME, Hagg PM, Kokkonen N, Vayrynen JP, Haapasaari KM, Moilanen J, Kallioinen M, Hurskainen T, Tasanen K (2016). Glucocorticoid receptors GRα and GRβ are expressed in inflammatory dermatoses. European Journal of Dermatology.

[ref-22] Kumar R, Johnson BH, Thompson EB (2004). Overview of the structural basis for transcription regulation by nuclear hormone receptors. Essays in Biochemistry.

[ref-23] Labrador L, Torres K, Camargo M, Santiago L, Valderrama E, Chiurillo MA (2015). Association of common variants on chromosome 8q24 with gastric cancer in Venezuelan patients. Gene.

[ref-70] Lee PH, Shatkay H (2008). F-SNP: computationally predicted functional SNPs for disease association studies. Nucleic Acids Research.

[ref-24] Leung DYM, Hamid Q, Vottero A, Szefler SJ, Surs W, Minshall E, Chrousos GP, Klemm DJ (1997). Association of glucocorticoid insensitivity with increased expression of glucocorticoid receptor beta. Journal of Experimental Medicine.

[ref-25] Li Q, Qu F, Li R, He X, Zhai Y, Chen W, Zheng Y (2017). A functional polymorphism of SSBP1 gene predicts prognosis and response to chemotherapy in resected gastric cancer patients. Oncotarget.

[ref-26] Lin Y, Ge Y, Wang Y, Ma G, Wang X, Liu H, Wang M, Zhang Z, Chu H (2017). The association of rs710886 in lncRNA PCAT1 with bladder cancer risk in a Chinese population. Gene.

[ref-27] Ling H, Spizzo R, Atlasi Y, Nicoloso M, Shimizu M, Redis RS, Nishida N, Gafa R, Song J, Guo Z, Ivan C, Barbarotto E, De Vries I, Zhang X, Ferracin M, Churchman M, Van Galen JF, Beverloo BH, Shariati M, Haderk F, Estecio MR, Garcia-Manero G, Patijn GA, Gotley DC, Bhardwaj V, Shureiqi I, Sen S, Multani AS, Welsh J, Yamamoto K, Taniguchi I, Song MA, Gallinger S, Casey G, Thibodeau SN, Le Marchand L, Tiirikainen M, Mani SA, Zhang W, Davuluri RV, Mimori K, Mori M, Sieuwerts AM, Martens JW, Tomlinson I, Negrini M, Berindan-Neagoe I, Foekens JA, Hamilton SR, Lanza G, Kopetz S, Fodde R, Calin GA (2013). CCAT2, a novel noncoding RNA mapping to 8q24, underlies metastatic progression and chromosomal instability in colon cancer. Genome Research.

[ref-28] Ma G, Gu D, Lv C, Chu H, Xu Z, Tong N, Wang M, Tang C, Xu Y, Zhang Z (2015). Genetic variant in 8q24 is associated with prognosis for gastric cancer in a Chinese population. Journal of Gastroenterology and Hepatology.

[ref-29] Marino JS, Stechschulte LA, Stec DE, Nestor-Kalinoski A, Coleman S, Hinds TD (2016). Glucocorticoid receptor β induces hepatic steatosis by augmenting inflammation and inhibition of the peroxisome proliferator-activated receptor (PPAR) α. Journal of Biological Chemistry.

[ref-30] Mcbeth L, Nwaneri AC, Grabnar M, Demeter J, Nestor-Kalinoski A, Hinds TD (2016). Glucocorticoid receptor beta increases migration of human bladder cancer cells. Oncotarget.

[ref-31] Mercer TR, Dinger ME, Mattick JS (2009). Long non-coding RNAs: insights into functions. Nature Reviews Genetics.

[ref-32] Ozgur E, Ferhatoglu F, Sen F, Saip P, Gezer U (2019). Circulating lncRNA H19 may be a useful marker of response to neoadjuvant chemotherapy in breast cancer. Cancer Biomark.

[ref-33] Pan W, Liu L, Wei J, Ge Y, Zhang J, Chen H, Zhou L, Yuan Q, Zhou C, Yang M (2016). A functional lncRNA HOTAIR genetic variant contributes to gastric cancer susceptibility. Molecular Carcinogenesis.

[ref-34] Patki M, McFall T (2018). Chronic p27Kip1 induction by dexamethasone causes senescence phenotype and permanent cell cycle blockade in lung adenocarcinoma cells over-expressing glucocorticoid receptor. Scientific Reports.

[ref-35] Prensner JR, Iyer MK, Balbin OA, Dhanasekaran SM, Cao Q, Brenner JC, Laxman B, Asangani IA, Grasso CS, Kominsky HD, Cao X, Jing X, Wang X, Siddiqui J, Wei JT, Robinson D, Iyer HK, Palanisamy N, Maher CA, Chinnaiyan AM (2011). Transcriptome sequencing across a prostate cancer cohort identifies PCAT-1, an unannotated lincRNA implicated in disease progression. Nature Biotechnology.

[ref-36] Psarra AMG, Solakidi S, Trougakos IP, Margaritis LH, Spyrou G, Sekeris CE (2005). Glucocorticoid receptor isoforms in human hepatocarcinoma HepG2 and SaOS-2 osteosarcoma cells: presence of glucocorticoid receptor alpha in mitochondria and of glucocorticoid receptor beta in nucleoli. International Journal of Biochemistry & Cell Biology.

[ref-37] Qu F, Chen Y, Wang X, He X, Ren T, Huang Q, Zhang J, Liu X, Guo X, Gu J, Xing J (2015). Leukocyte mitochondrial DNA content: a novel biomarker associated with prognosis and therapeutic outcome in colorectal cancer. Carcinogenesis.

[ref-38] Ren Y, Shang J, Li J, Liu W, Zhang Z, Yuan J, Yang M (2017). The long noncoding RNA PCAT-1 links the microRNA miR-215 to oncogene CRKL-mediated signaling in hepatocellular carcinoma. Journal of Biological Chemistry.

[ref-39] Saeki N, Ono H, Sakamoto H, Yoshida T (2013). Genetic factors related to gastric cancer susceptibility identified using a genome-wide association study. Cancer Science.

[ref-40] Sakamoto H, Yoshimura K, Saeki N, Katai H, Shimoda T, Matsuno Y, Saito D, Sugimura H, Tanioka F, Kato S (2008). Genetic variation in PSCA is associated with susceptibility to diffuse-type gastric cancer. Nature Genetics.

[ref-41] Sattarifard H, Hashemi M, Hassanzarei S, Narouie B, Bahari G (2017). Association between genetic polymorphisms of long non-coding RNA PRNCR1 and prostate cancer risk in a sample of the Iranian population. Molecular and Clinical Oncology.

[ref-42] Shi Y, Hu Z, Wu C, Dai J, Li H, Dong J, Wang M, Miao X, Zhou Y, Lu F, Zhang H, Hu L, Jiang Y, Li Z, Chu M, Ma H, Chen J, Jin G, Tan W, Wu T, Zhang Z, Lin D, Shen H (2011). A genome-wide association study identifies new susceptibility loci for non-cardia gastric cancer at 3q13.31 and 5p13.1. Nature Genetics.

[ref-43] Shi WH, Wu QQ, Li SQ, Yang TX, Liu ZH, Tong YS, Tuo L, Wang S, Cao XF (2015). Upregulation of the long noncoding RNA PCAT-1 correlates with advanced clinical stage and poor prognosis in esophageal squamous carcinoma. Tumor Biology.

[ref-44] Silvestri V, Rizzolo P, Scarno M, Chillemi G, Navazio AS, Valentini V, Zelli V, Zanna I, Saieva C, Masala G, Bianchi S, Manoukian S, Barile M, Pensotti V, Peterlongo P, Varesco L, Tommasi S, Russo A, Giannini G, Cortesi L, Viel A, Montagna M, Radice P, Palli D, Ottini L (2015). Novel and known genetic variants for male breast cancer risk at 8q24.21, 9p21.3, 11q13.3 and 14q24.1: results from a multicenter study in Italy. European Journal of Cancer.

[ref-45] Song P, Jiang B, Liu Z, Ding J, Liu S, Guan W (2017). A three-lncRNA expression signature associated with the prognosis of gastric cancer patients. Cancer Medicine.

[ref-46] Sur I, Tuupanen S, Whitington T, Aaltonen LA, Taipale J (2013). Lessons from functional analysis of genome-wide association studies. Cancer Research.

[ref-47] Tong Y, Wang H, Li S, Zhao F, Ying J, Qu Y, Mu D (2018). Cumulative evidence for relationships between multiple variants in 8q24 and colorectal cancer incidence. Medicine.

[ref-48] Ülger Y, Dadaş E,  Yalinbaş Kaya B, Sümbül AT,  Genç A,  Bayram S (2017). The analysis of lncRNA HOTAIR rs12826786 C>T polymorphism and gastric cancer susceptibility in a Turkish population: lack of any association in a hospital-based case-control study. Irish Journal of Medical Science.

[ref-49] Ulrich CM, Robien K, McLeod HL (2003). Cancer pharmacogenetics: polymorphisms, pathways and beyond. Nature Reviews Cancer.

[ref-50] Wadhwa R, Song S, Lee JS, Yao Y, Wei Q, Ajani JA (2013). Gastric cancer—molecular and clinical dimensions. Nature Reviews Clinical Oncology.

[ref-51] Wang W, Du M, Li Z, Zhang L, Li Q, Xu Z, Li B, Wang L, Li F, Zhang D, Xu H, Yang L, Gong W, Qiang F, Zhang Z, Xu Z (2018). A genetic variant located in miR-146b promoter region is associated with prognosis of gastric cancer. Cancer Epidemiology Biomarkers & Prevention.

[ref-52] Wang X, Liu Y, Shao D, Qian Z, Dong Z, Sun Y, Xing X, Cheng X, Du H, Hu Y, Li Y, Li L, Dong B, Li Z, Wu A, Wu X, Bu Z, Zong X, Zhu G, Ji Q, Wen XZ, Zhang LH, Ji JF (2016). Recurrent amplification of MYC and TNFRSF11B in 8q24 is associated with poor survival in patients with gastric cancer. Gastric Cancer.

[ref-53] Wang X, Wang X (2019). Long non-coding RNA colon cancer-associated transcript 2 may promote esophageal cancer growth and metastasis by regulating the Wnt signaling pathway. Oncology Letters.

[ref-54] Washington K (2010). 7th Edition of the AJCC cancer staging manual: stomach. Annals of Surgical Oncology.

[ref-55] Weidle UH, Birzele F, Kollmorgen G, Ruger R (2017). Long Non-coding RNAs and their Role in Metastasis. Cancer Genomics & Proteomics.

[ref-56] Wu ER, Hsieh MJ (2019). Association of lncRNA CCAT2 and CASC8 gene polymorphisms with hepatocellular carcinoma. International Journal of Environmental Research and Public Health.

[ref-57] Xiang J-F, Yin Q-F, Chen T, Zhang Y, Zhang X-O, Wu Z, Zhang S, Wang H-B, Ge J, Lu X, Yang L, Chen L-L (2014). Human colorectal cancer-specific CCAT1-L lncRNA regulates long-range chromatin interactions at the MYC locus. Cell Research.

[ref-58] Xiong Y, Wang R, Peng L, You W, Wei J, Zhang S, Wu X, Guo J, Xu J, Lv Z (2017). An integrated lncRNA, microRNA and mRNA signature to improve prognosis prediction of colorectal cancer. Oncotarget.

[ref-59] Yang ML, Huang Z, Wu LN, Wu R, Ding HX, Wang BG (2019). lncRNA-PCAT1 rs2632159 polymorphism could be a biomarker for colorectal cancer susceptibility. Bioscience Reports.

[ref-60] Yemelyanov A, Czwornog J, Chebotaev D, Karseladze A, Kulevitch E, Yang X, Budunova I (2006). Tumor suppressor activity of glucocorticoid receptor in the prostate. Oncogene.

[ref-61] Ying Y, Zhang X, Li Z, Deng L, Jiao G, Zhang B, Ping X, Mu H, Qiao W, Jian Z (2013). Glucocorticoid receptor β regulates injury-mediated astrocyte activation and contributes to glioma pathogenesis via modulation of β-catenin/TCF transcriptional activity. Neurobiology of Disease.

[ref-62] Yuan Q, Chu H, Ge Y, Ma G, Du M, Wang M, Zhang Z, Zhang W (2018). LncRNA PCAT1 and its genetic variant rs1902432 are associated with prostate cancer risk. Journal of Cancer.

[ref-63] Zeichner SB, Goldstein DA, Kohn C, Flowers CR (2017). Cost-effectiveness of precision medicine in gastrointestinal stromal tumor and gastric adenocarcinoma. Journal of Gastrointestinal Oncology.

[ref-64] Zhang M, Du X (2016). Noncoding RNAs in gastric cancer: research progress and prospects. World Journal of Gastroenterology.

[ref-65] Zhao B, Hou X, Zhan H (2015). Long non-coding RNA PCAT-1 over-expression promotes proliferation and metastasis in non-small cell lung cancer cells. International Journal of Clinical and Experimental Medicine.

[ref-66] Zhao J, Liu Y, Huang G, Cui P, Zhang W, Zhang Y (2015). Long non-coding RNAs in gastric cancer: versatile mechanisms and potential for clinical translation. American Journal of Cancer Research.

[ref-67] Zheng Y, Izumi K, Li Y, Ishiguro H, Miyamoto H (2012). Contrary regulation of bladder cancer cell proliferation and invasion by dexamethasone-mediated glucocorticoid receptor signals. Molecular Cancer Therapeutics.

[ref-68] Zhi P, Shi J, Liu F (2017). Genetic variations at 8q24 and gastric cancer susceptibility: a meta-analysis study. PLOS ONE.

[ref-69] Zhu J, Gong JY, Goodman OB, Cartegni L, Nanus DM, Shen R (2007). Bombesin attenuates pre-mRNA splicing of glucocorticoid receptor by regulating the expression of serine-arginine protein p30c (SRp30c) in prostate cancer cells. Biochimica et Biophysica Acta (BBA) - Molecular Cell Research.

